# Modified Projection Algorithms for Solving the Split Equality Problems

**DOI:** 10.1155/2014/328787

**Published:** 2014-01-19

**Authors:** Qiao-Li Dong, Songnian He

**Affiliations:** College of Science, Civil Aviation University of China, Tianjin 300300, China

## Abstract

The split equality problem (SEP) has extraordinary utility and broad applicability in many areas of applied mathematics. Recently, Byrne and Moudafi (2013) proposed a CQ algorithm for solving it. In this paper, we propose a modification for the CQ algorithm, which computes the stepsize adaptively and performs an additional projection step onto two half-spaces in each iteration. We further propose a relaxation scheme for the self-adaptive projection algorithm by using projections onto half-spaces instead of those onto the original convex sets, which is much more practical. Weak convergence results for both algorithms are analyzed.

## 1. Introduction

The split equality problem (SEP) was introduced by Moudafi [1] and its interest covers many situations, for instance, in domain decomposition for PDE's, game theory, and intensity-modulated radiation therapy (IMRT) (see [2–7] for more details). Let *H*_1_, *H*_2_, and *H*_3_ be real Hilbert spaces; let *C* ⊂ *H*_1_ and *Q* ⊂ *H*_2_ be two nonempty closed convex sets; let *A* : *H*_1_ → *H*_3_ and *B* : *H*_2_ → *H*_3_ be two bounded linear operators. The SEP can mathematically be formulated as the problem of finding *x* and *y* with the property

(1) $$\begin{array}{c} x\in C,\;y\in Q,\;\text{such}\;\text{that}\;Ax=By, \end{array}$$

which allows asymmetric and partial relations between the variables *x* and *y*. If *H*_2_ = *H*_3_ and *B* = *I*, then the split equality problem (1) reduces to the split feasibility problem (originally introduced in Censor and Elfving [8]) which is to find *x* ∈ *C* with *Ax* ∈ *Q*.

For solving the SEP (1), Moudafi [1] introduced the following alternating *CQ* algorithm:

(2) $$\begin{array}{c} x_{k+1}=P_{C}\left(x_{k}-\gamma_{k}A^{*}\left(Ax_{k}-By_{k}\right)\right),\\ y_{k+1}=P_{Q}\left(y_{k}+\gamma_{k}B^{*}\left(Ax_{k+1}-By_{k}\right)\right), \end{array}$$

where *γ*_*k*_ ∈ (*ɛ*, min(1/*λ*_*A*_, 1/*λ*_*B*_) − *ɛ*) and *λ*_*A*_ and *λ*_*B*_ are the spectral radii of A*A and B*B, respectively. By studying the projected Landweber algorithm of the SEP (1) in a product space, Byrne and Moudafi [7] obtained the following *CQ* algorithm:

(3) $$\begin{array}{c} x_{k+1}=P_{C}\left(x_{k}-\gamma_{k}A^{*}\left(Ax_{k}-By_{k}\right)\right),\\ y_{k+1}=P_{Q}\left(y_{k}+\gamma_{k}B^{*}\left(Ax_{k}-By_{k}\right)\right), \end{array}$$

where *γ*_*k*_, the stepsize at the iteration *k*, is chosen in the interval  (*ɛ*, (2/(*λ*_*A*_ + *λ*_*B*_)) − *ɛ*). It is easy to see that the alternating *CQ* algorithm (2) is sequential but the algorithm (3) is simultaneous.

Observe that in the algorithms (2) and (3), the determination of the stepsize *γ*_*n*_ depends on the operator (matrix) norms ||*A*|| and ||*B*|| (or the largest eigenvalues of A*A and B*B). This means that, in order to implement the alternating *CQ* algorithm (2), one has first to compute (or, at least, estimate) operator norms of *A* and *B*, which is in general not an easy work in practice. Considering this, Dong and He [9] proposed algorithms without prior knowledge of operator norms.

In this paper, we first propose a modification for *CQ* algorithm (3), inspired by Tseng [10] (also see [11]). Our modified projection method computes the stepsize adaptively and performs an additional projection step onto two half-spaces, *X*_*k*_ ⊂ *H*_1_ and *Y*_*k*_ ⊂ *H*_2_, in each iteration. Then we give a relaxation scheme for this modification by replacing the orthogonal projections onto the sets *C* and *Q* by projections onto the two half-spaces *C*_*k*_ and *Q*_*k*_, respectively. Since projections onto half-spaces can be directly calculated, the relaxed scheme will be more practical and easily implemented.

The rest of this paper is organized as follows. In the next section, some useful facts and tools are given. The weak theorem of the proposed self-adaptive projection algorithm is obtained in Section 3. In Section 4, we consider a relaxed self-adaptive projection algorithm, where the sets *C* and *Q* are level sets of convex functions.

## 2. Preliminaries

In this section, we review some definitions and lemmas which will be used in this paper.

Let *H* be a Hilbert space and let *I* be the identity operator on *H*. If *f* : *H* → ℝ is a differentiable functional, then denote by ∇*f* the gradient of *f*. If *f* : *H* → ℝ is a subdifferentiable functional, then denote by ∂*f* the subdifferential of *f*. Given a sequence (*x*_*k*_, *y*_*k*_) in *H*_1_ × *H*_2_, *ω*_*w*_(*x*_*k*_, *y*_*k*_) stands for the set of cluster points in the weak topology. “*x*_*k*_ → *x*” (resp., “*x*_*k*_⇀*x*”) means the strong (resp., weak) convergence of (*x*_*k*_) to *x*.

**Definition**

A sequence (*x*_*k*_) is said to be asymptotically regular if

(4) $$\begin{array}{c} \underset{k\to \infty }{\lim}||x_{k+1}-x_{k}||=0. \end{array}$$

**Definition 2**

The graph of an operator is called to be weakly-strongly closed if *y*_*n*_ ∈ *T*(*x*_*n*_) with *y*_*n*_ strongly converging to *y* and *x*_*n*_ weakly converging to *x*; then *y* ∈ *T*(*x*).

The next lemma is well known (see [10, 12]) and shows that the maximal monotone operators are weakly-strongly closed.

**Lemma 3**

Let *H* be a Hilbert space and let *T* : *H*⇉*H* be a maximal monotone mapping. If (*x*_*k*_) is a sequence in *H* bounded in norm and converging weakly to some *x* and (*w*_*k*_) is a sequence in *H* converging strongly to some *w* and *w*_*k*_ ∈ *T*(*x*_*k*_) for all *k*, then *w* ∈ *T*(*x*).

The projection is an important tool for our work in this paper. Let *Ω* be a closed convex subset of real Hilbert space *H*. Recall that the (nearest point or metric) projection from *H* onto *Ω*, denoted by  *P*_*Ω*_, is defined in such a way that, for each *x* ∈ *H*, *P*_*Ω*_ *x* is the unique point in *Ω* such that

(5) $$\begin{array}{c} ||x-P_{\Omega }x||=\min\left\{||x-z||:z\in \Omega \right\}. \end{array}$$

The following two lemmas are useful characterizations of projections.

**Lemma 4**

Given *x* ∈ *H* and *z* ∈ *Ω*, then *z* = *P*_*Ω*_ *x* if and only if

(6) $$\begin{array}{c} \langle x-z,y-z\rangle \leq 0,\;\forall y\in \Omega . \end{array}$$

**Lemma 5**

For any *x*, *y* ∈ *H* and *z* ∈ *Ω*, it holds

- ||*P*_*Ω*_(*x*)−*P*_*Ω*_(*y*)||^2^ ≤ 〈*P*_*Ω*_(*x*) − *P*_*Ω*_(*y*), *x* − *y*〉;
- ||*P*_*Ω*_(*x*)−*z*||^2^ ≤ ||*x*−*z*||^2^ − ||*P*_*Ω*_(*x*)−*x*||^2^.

Throughout this paper, assume that the split equality problem (1) is consistent and denote by Γ the solution of (1); that is,

(7) $$\begin{array}{c} \Gamma =\left\{x\in C,y\in Q:Ax=By\right\}. \end{array}$$

Then Γ is closed, convex, and nonempty. The split equality problem (1) can be written as the following minimization problem:

(8) $$\begin{array}{c} \underset{x\in H_{1},\;y\in H_{2}}{\min}\iota_{C}\left(x\right)+\iota_{Q}\left(y\right)+\frac{1}{2}{||Ax-By||}^{2}, \end{array}$$

where *ι*_*C*_(*x*) is an indicator function of the set *C* defined by

(9) $$\begin{array}{c} \iota_{C}\left(x\right)=\{\begin{array}{cc} 0, & x\in C\\ +\infty , & \text{otherwise}. \end{array} \end{array}$$

By writing down the optimality conditions, we obtain

(10) $$\begin{array}{c} 0\in \nabla_{x}\left\{\frac{1}{2}{||Ax-By||}^{2}\right\}+\partial \iota_{C}\left(x\right)=A^{*}\left(Ax-By\right)+N_{C}\left(x\right),\\ 0\in \nabla_{y}\left\{\frac{1}{2}{||Ax-By||}^{2}\right\}+\partial \iota_{Q}\left(y\right)=-B^{*}\left(Ax-By\right)+N_{Q}\left(y\right), \end{array}$$

which implies, for *γ* > 0 and *β* > 0,

(11) $$\begin{array}{c} x-\gamma A^{*}\left(Ax-By\right)\in x+\gamma N_{C}\left(x\right),\\ y+\beta B^{*}\left(Ax-By\right)\in y+\beta N_{Q}\left(y\right), \end{array}$$

which in turn leads to the fixed point formulation

(12) $$\begin{array}{c} x={\left(I+\gamma N_{C}\right)}^{-1}\left(x-\gamma A^{*}\left(Ax-By\right)\right),\\ y={\left(I+\beta N_{Q}\right)}^{-1}\left(y+\beta B^{*}\left(Ax-By\right)\right). \end{array}$$

Since (*I* + *γN*_*C*_)^−1^ = *P*_*C*_ and (*I* + *βN*_*Q*_)^−1^ = *P*_*Q*_, we have

(13) $$\begin{array}{c} x=P_{C}\left(x-\gamma A^{*}\left(Ax-By\right)\right),\\ y=P_{Q}\left(y+\beta B^{*}\left(Ax-By\right)\right). \end{array}$$

The following proposition shows that solutions of the fixed point equations (17) are exactly the solutions of the SEP (1).

**Proposition (see [9])**

Given x* ∈ *H*_1_ and y* ∈ *H*_2_, then (x*, y*) solves the SEP (1) if and only if (x*, y*) solves the fixed point equations (13).

## 3. A Self-Adaptive Projection Algorithm

Based on Proposition 6, we construct a self-adaptive projection algorithm for the fixed point equations (13) and prove the weak convergence of the proposed algorithm.

Define the function *F* : *H*_1_ × *H*_2_ → *H*_1_ by

(14) $$\begin{array}{c} F\left(x,y\right)=A^{*}\left(Ax-By\right) \end{array}$$

and the function *G* : *H*_1_ × *H*_2_ → *H*_2_ by

(15) $$\begin{array}{c} G\left(x,y\right)=B^{*}\left(By-Ax\right). \end{array}$$

The self-adaptive projection algorithm is defined as follows.

**Algorithm 7**

Given constants *σ*_0_ > 0,  *β* ∈ (0,1),  *θ* ∈ (0,1) and *ρ* ∈ (0,1), let *x*_0_ ∈ *H*_1_ and *y*_0_ ∈ *H*_2_ be arbitrary. For *k* = 0,1, 2,…, compute

(16) $$\begin{array}{c} u_{k}=P_{C}\left(x_{k}-\tau_{k}F\left(x_{k},y_{k}\right)\right),\\ v_{k}=P_{Q}\left(y_{k}-\tau_{k}G\left(x_{k},y_{k}\right)\right), \end{array}$$

where *γ*_*k*_ is chosen to be the largest *γ* ∈ {*σ*_*k*_, *σ*_*k*_ *β*, *σ*_*k*_ *β* ^2^,…} satisfying

(17) ||F(xk,yk)−F(uk,vk)||2+||G(xk,yk)−G(uk,vk)||2  ≤θ2||xk−uk||2+||yk−vk||2γ2.

Construct the half-spaces *X*_*k*_ and *Y*_*k*_, the bounding hyperplanes of which support *C* and *Q* at *u*_*k*_ and *v*_*k*_, respectively,

(18) $$\begin{array}{c} X_{k}∶=\left\{u\in H_{1}\;|\;\langle x_{k}-\tau_{k}F\left(x_{k},y_{k}\right)-u_{k},u-u_{k}\rangle \leq 0\right\},\\ Y_{k}∶=\left\{v\in H_{2}\;|\;\langle y_{k}-\tau_{k}G\left(x_{k},y_{k}\right)-v_{k},v-v_{k}\rangle \leq 0\right\}. \end{array}$$

Set

(19) $$\begin{array}{c} x_{k+1}=P_{X_{k}}\left(u_{k}-\gamma_{k}\left(F\left(u_{k},v_{k}\right)-F\left(x_{k},y_{k}\right)\right)\right),\\ y_{k+1}=P_{Y_{k}}\left(v_{k}-\gamma_{k}\left(G\left(u_{k},v_{k}\right)-G\left(x_{k},y_{k}\right)\right)\right). \end{array}$$

If

(20) ||F(xk+1,yk+1)−F(xk,yk)||2+||G(xk+1,yk+1)−G(xk,yk)||2  ≤ρ2||xk+1−xk||2+||yk+1−yk||2γk2,

then set *σ*_*k*_ = *σ*_0_; otherwise, set *σ*_*k*_ = *γ*_*k*_.

In this algorithm, (19) involves projection onto half-spaces *X*_*k*_ (resp., *Y*_*k*_) rather than onto the set *C* (resp., *Q*) and it is obvious that projections on *X* (resp., *Y*) are very simple. It is easy to show *C* ⊂ *X*_*k*_ and *Q* ⊂ *Y*_*k*_. The last step is used to reduce the inner iterations for searching the stepsize *γ*_*k*_.

**Lemma 8**

The search rule (17) is well defined. Besides $\underline{\gamma }\leq \gamma_{k}\leq \sigma_{0}$, where

(21) γ_=min{σ0,βθ||A||2(||A||2+||B||2),   βθ||B||2(||A||2+||B||2)}.

**Proof**

Obviously, *γ*_*k*_ ≤ *σ*_0_. If *γ*_*k*_ = *σ*_0_, then this lemma is proved; otherwise, if *γ*_*k*_ < *σ*_0_, by the search rule (17), we know that *γ*_*k*_/*β* must violate inequality (17); that is,

(22) ||F(xk,yk)−F(uk,vk)||2+||G(xk,yk)−G(uk,vk)||2  ≥θ2||xk−uk||2+||yk−vk||2γk2/β2.

On the other hand, we have

(23) ||F(xk,yk)−F(uk,vk)||2+||G(xk,yk)−G(uk,vk)||2 =||A∗(Axk−Byk)−A∗(Auk−Bvk)||2  +||B∗(Byk−Axk)−B∗(Bvk−Auk)||2 ≤(||A||2+||B||2)  ×(||A||||xk−uk||+||B||||yk−vk||)2 ≤2(||A||2+||B||2)  ×(||A||2||xk−uk||2+||B||2||yk−vk||2) ≤2(||A||2+||B||2)max{||A||2,||B||2}  ×(||xk−uk||2+||yk−vk||2).

Consequently, we get

(24) γk≥min{σ0,βθ||A||2(||A||2+||B||2),   βθ||B||2(||A||2+||B||2)},

which completes the proof.

**Theorem 9**

Let (*x*_*k*_, *y*_*k*_) be the sequence generated by Algorithm 7 and let *X* and *Y* be nonempty closed convex sets in *H*_1_ and *H*_2_ with simple structures, respectively. If (*X* × *Y*)∩Γ is nonempty, then (*x*_*k*_, *y*_*k*_) converges weakly to a solution of the SEP (1).

**Proof**

Let (x*, y*) ∈ Γ; that is, x* ∈ *C*, y* ∈ *Q*, and Ax* = By*. Define *s*_*k*_ = *u*_*k*_ − *γ*_*k*_(*F*(*u*_*k*_, *v*_*k*_) − *F*(*x*_*k*_, *y*_*k*_)); then we have

(25) ||xk+1−x∗||2 ≤||sk−x∗||2 =||sk−uk+uk−xk+xk−x∗||2 =||sk−uk||2+||uk−xk||2  +||xk−x∗||2+2〈sk−uk,uk−x∗〉  +2〈uk−xk,xk−x∗〉 =||xk−x∗||2+||sk−uk||2−||uk−xk||2  +2〈sk−xk,uk−x∗〉 =||xk−x∗||2+γk2||F(uk,vk)−F(xk,yk)||2  −||uk−xk||2+2〈sk−xk,uk−x∗〉,

where the first inequality follows from nonexpansivity of the projection mapping *P*_*X*_*k*__. Similarly, defining *t*_*k*_ = *v*_*k*_ − *γ*_*k*_(*G*(*u*_*k*_, *v*_*k*_) − *G*(*x*_*k*_, *y*_*k*_)), we get

(26) ||yk+1−y∗||2≤||yk−y∗||2 +γk2||G(uk,vk)−G(xk,yk)||2 −||vk−yk||2+2〈tk−yk,vk−y∗〉.

Adding the above inequalities, we obtain

(27) ||xk+1−x∗||2+||yk+1−y∗||2 ≤||xk−x∗||2+||yk−y∗||2  +γk2(||F(uk,vk)−F(xk,yk)||2      +||G(uk,vk)−G(xk,yk)||2)  −||uk−xk||2−||vk−yk||2  +2〈sk−xk,uk−x∗〉+2〈tk−yk,vk−y∗〉 ≤||xk−x∗||2+||yk−y∗||2−(1−θ2)  ×(||uk−xk||2+||vk−yk||2)  +2〈sk−xk,uk−x∗〉+2〈tk−yk,vk−y∗〉 =||xk−x∗||2+||yk−y∗||2−(1−θ2)  ×(||uk−xk||2+||vk−yk||2)  +2〈uk−γkF(uk,vk)     +γkF(xk,yk)−xk,uk−x∗〉  +2〈vk−γkG(uk,vk)     +γkG(xk,yk)−yk,vk−y∗〉 ≤||xk−x∗||2+||yk−y∗||2  −(1−θ2)(||uk−xk||2+||vk−yk||2)  −2γk〈F(uk,vk),uk−x∗〉  −2γk〈G(uk,vk),vk−y∗〉,

where the equality follows from *s*_*k*_ = *u*_*k*_ − *γ*_*k*_(*F*(*u*_*k*_, *v*_*k*_) − *F*(*x*_*k*_, *y*_*k*_)) and *t*_*k*_ = *v*_*k*_ − *γ*_*k*_(*G*(*u*_*k*_, *v*_*k*_) − *G*(*x*_*k*_, *y*_*k*_)), the second inequality follows from (17), and the last follows from (16) and Lemma 3 and x* ∈ *C*,  y* ∈ *Q*. Using the fact and Ax* = By*, we have

(28) 〈F(uk,vk),uk−x∗〉+〈G(uk,vk),vk−y∗〉 =〈A∗(Auk−Bvk),uk−x∗〉  +〈B∗(Bvk−Auk),vk−y∗〉 =〈Auk−Bvk,Auk−Ax∗〉  +〈Bvk−Auk,Bvk−By∗〉 =||Auk−Bvk||2,

which with (27) implies that

(29) ||xk+1−x∗||2+||yk+1−y∗||2 ≤||xk−x∗||2+||yk−y∗||2−(1−θ2)  ×(||uk−xk||2+||vk−yk||2)  −2γk||Auk−Bvk||2.

Consequently, the sequence Γ_*k*_(x*, y*)∶ = ||*x*_*k*_−x*||^2^ + ||*y*_*k*_−y*||^2^ is decreasing and lower bounded by 0 and thus converges to some finite limit, say, *l*(x*, y*). Moreover, (*x*_*k*_) and (*y*_*k*_) are bounded. This implies that

(30) $$\begin{array}{c} \underset{k\to \infty }{\lim}||u_{k}-x_{k}||=0,\;\;\underset{k\to \infty }{\lim}||v_{k}-y_{k}||=0,\\ \underset{k\to \infty }{\lim}||Au_{k}-Bv_{k}||=0. \end{array}$$

From (30), we get

(31) $$\begin{array}{c} \underset{k\to \infty }{\lim}||Ax_{k}-By_{k}||=0. \end{array}$$

Let $\left(\hat{x},\hat{y})\in \omega_{w}(x_{k},y_{k}\right)$; then there exist the two subsequences (*x*_*k*_*l*__) and (*y*_*k*_*l*__) of (*x*_*k*_) and (*y*_*k*_) which converge weakly to $\hat{x}$ and $\hat{y}$, respectively. We will show that $\left(\hat{x},\hat{y}\right)$ is a solution of the SEP (1). The weak convergence of (*Ax*_*k*_*l*__ − *By*_*k*_*l*__) to $A\hat{x}-B\hat{y}$ and lower semicontinuity of the squared norm imply that

(32) $$\begin{array}{c} ||A\hat{x}-B\hat{y}||\leq \underset{l\to \infty }{\mathrm{liminf}}||Ax_{k_{l}}-By_{k_{l}}||=0; \end{array}$$

that is, $A\hat{x}=B\hat{y}$.

By noting that the two equalities in (16) can be rewritten as

(33) $$\begin{array}{c} \frac{x_{k_{l}}-u_{k_{l}}}{\gamma_{k_{l}}}-A^{*}\left(Au_{k_{l}}-Bv_{k_{l}}\right)\in N_{C}\left(u_{k_{l}}\right),\\ \frac{y_{k_{l}}-v_{k_{l}}}{\gamma_{k_{l}}}-B^{*}\left(Bv_{k_{l}}-Au_{k_{l}}\right)\in N_{Q}\left(v_{k_{l}}\right), \end{array}$$

and that the graphs of the maximal monotone operators, *N*_*C*_ and *N*_*Q*_, are weakly-strongly closed and by passing to the limit in the last inclusions, we obtain, from (30), that

(34) $$\begin{array}{c} \hat{x}\in C,\;\hat{y}\in Q. \end{array}$$

Hence $(\hat{x},\hat{y})\in \Gamma$.

To show the uniqueness of the weak cluster points, we will use the same strick as in the celebrated Opial Lemma. Indeed, let $\left(\bar{x},\bar{y}\right)$ be other weak cluster point of (*x*_*k*_, *y*_*k*_). By passing to the limit in the relation

(35) Γk(x^,y^)=Γk(x¯,y¯)+||x^−x¯||2+||y^−y¯||2 +2〈xk−x¯,x¯−x^〉+2〈yk−y¯,y¯−y^〉,

we obtain

(36) $$\begin{array}{c} l\left(\hat{x},\hat{y}\right)=l\left(\bar{x},\bar{y}\right)+{||\hat{x}-\bar{x}||}^{2}+{||\hat{y}-\bar{y}||}^{2}. \end{array}$$

Reversing the role of $\left(\hat{x},\hat{y}\right)$ and $\left(\bar{x},\bar{y}\right)$, we also have

(37) $$\begin{array}{c} l\left(\bar{x},\bar{y}\right)=l\left(\hat{x},\hat{y}\right)+{||\hat{x}-\bar{x}||}^{2}+{||\hat{y}-\bar{y}||}^{2}. \end{array}$$

By adding the two last equalities, we obtain

(38) $$\begin{array}{c} {||\hat{x}-\bar{x}||}^{2}+{||\hat{y}-\bar{y}||}^{2}=0. \end{array}$$

Hence $\left(\hat{x},\hat{y})=(\bar{x},\bar{y}\right)$; this implies that the whole sequence (*x*_*k*_, *y*_*k*_) weakly converges to a solution of the SEP (1), which completes the proof.

## 4. A Relaxed Self-Adaptive Projection Algorithm

In Algorithm 7, we must calculate the orthogonal projections, *P*_*C*_ and *P*_*Q*_, many times even in one iteration step, so they should be assumed to be easily calculated; however, sometimes it is difficult or even impossible to compute them. In this case, we always turn to relaxed methods [13, 14], which were introduced by Fukushima [15] and are more easily implemented. For solving the SEP (1), Moudafi [16] followed the ideas of Fukushima [15] and introduced a relaxed alternating *CQ* algorithm which depends on the norms ||*A*|| and ||*B*||. In this section, we propose a relaxed scheme for the self-adaptive Algorithm 7.

Assume that the convex sets *C* and *Q* are given by

(39) $$\begin{array}{c} C=\left\{x\in H_{1}:c\left(x\right)\leq 0\right\},\;\;Q=\left\{y\in H_{2}:q\left(y\right)\leq 0\right\}, \end{array}$$

where *c* : *H*_1_ → ℝ and *q* : *H*_2_ → ℝ are convex functions which are subdifferentiable on *C* and *Q*, respectively, and we assume that their subdifferentials are bounded on bounded sets.

In the *k*th iteration, let (*C*_*k*_) and (*Q*_*k*_) be two sequences of closed convex sets defined by

(40) $$\begin{array}{c} C_{k}=\left\{x\in H_{1}:c\left(x_{k}\right)+\langle \xi_{k},x-x_{k}\rangle \leq 0\right\}, \end{array}$$

where *ξ*_*k*_ ∈ ∂*c*(*x*_*k*_) and

(41) $$\begin{array}{c} Q_{k}=\left\{y\in H_{2}:q\left(y_{k}\right)+\langle \eta_{k},y-y_{k}\rangle \leq 0\right\}, \end{array}$$

where *η*_*k*_ ∈ ∂*q*(*y*_*k*_).

It is easy to see that *C*_*k*_⊃*C* and *Q*_*k*_⊃*Q* for every *k* ≥ 0.

**Algorithm 10**

Given constants *σ*_0_ > 0,  *β* ∈ (0,1),  *θ* ∈ (0,1),  and *ρ* ∈ (0,1), let *x*_0_ ∈ *H*_1_ and *y*_0_ ∈ *H*_2_ be arbitrary. For *k* = 0,1, 2,…, compute

(42) $$\begin{array}{c} u_{k}=P_{C_{k}}\left(x_{k}-\tau_{k}F\left(x_{k},y_{k}\right)\right),\\ v_{k}=P_{Q_{k}}\left(y_{k}-\tau_{k}G\left(x_{k},y_{k}\right)\right), \end{array}$$

where *γ*_*k*_ is chosen to be the largest *γ* ∈ {*σ*_*k*_, *σ*_*k*_ *β*, *σ*_*k*_ *β* ^2^,…} satisfying

(43) ||F(xk,yk)−F(uk,vk)||2  +||G(xk,yk)−G(uk,vk)||2 ≤θ2||xk−uk||2+||yk−vk||2γ2.

Construct the half-spaces *X*_*k*_ and *Y*_*k*_ the bounding hyperplanes of which support *C*_*k*_ and *Q*_*k*_ at *u*_*k*_ and *v*_*k*_, respectively,

(44) $$\begin{array}{c} X_{k}∶=\left\{u\in H_{1}\;|\;\langle x_{k}-\tau_{k}F\left(x_{k},y_{k}\right)-u_{k},u-u_{k}\rangle \leq 0\right\},\\ Y_{k}∶=\left\{v\in H_{2}\;|\;\langle y_{k}-\tau_{k}G\left(x_{k},y_{k}\right)-v_{k},v-v_{k}\rangle \leq 0\right\}. \end{array}$$

Set

(45) $$\begin{array}{c} x_{k+1}=P_{X}\left(u_{k}-\gamma_{k}\left(F\left(u_{k},v_{k}\right)-F\left(x_{k},y_{k}\right)\right)\right),\\ y_{k+1}=P_{Y}\left(v_{k}-\gamma_{k}\left(G\left(u_{k},v_{k}\right)-G\left(x_{k},y_{k}\right)\right)\right). \end{array}$$

If

(46) ||F(xk+1,yk+1)−F(xk,yk)||2  +||G(xk+1,yk+1)−G(xk,yk)||2 ≤ρ2||xk+1−xk||2+||yk+1−yk||2γk2,

then set *σ*_*k*_ = *σ*_0_; otherwise, set *σ*_*k*_ = *γ*_*k*_.

Following the proof of Lemma 8, we easily obtain the following.

**Lemma 11**

The search rule (43) is well defined. Besides $\underline{\gamma }\leq \gamma_{k}\leq \sigma_{0}$, where

(47) γ_=min{σ0,βθ||A||2(||A||2+||B||2),   βθ||B||2(||A||2+||B||2)}.

**Theorem 12**

Let (*x*_*k*_, *y*_*k*_) be the sequence generated by Algorithm 10 and let *X* and *Y* be nonempty closed convex sets in *H*_1_ and *H*_2_ with simple structures, respectively. If (*X* × *Y*)∩Γ is nonempty, then (*x*_*k*_, *y*_*k*_) converges weakly to a solution of the SEP (1).

**Proof**

Let (x*, y*) ∈ Γ; that is, x* ∈ *C*, y* ∈ *Q*, and Ax* = By*. Following the similar proof of Theorem 9, we obtain

(48) ||xk+1−x∗||2+||yk+1−y∗||2 ≤||xk−x∗||2+||yk−y∗||2  −(1−θ2)(||uk−xk||2+||vk−yk||2)  −2γk||Auk−Bvk||2.

Let Γ_*k*_(x*, y*)∶ = ||*x*_*k*_−x*||^2^ + ||*y*_*k*_−y*||^2^. Then the sequence Γ_*k*_(x*, y*) is decreasing and lower bounded by 0 for that *μ* ∈ (0,1) and thus converges to some finite limit, say, *l*(x*, y*). Moreover, (*x*_*k*_) and (*y*_*k*_) are bounded. This implies that

(49) $$\begin{array}{c} \underset{k\to \infty }{\lim}||u_{k}-x_{k}||=0,\;\;\underset{k\to \infty }{\lim}||v_{k}-y_{k}||=0, \end{array}$$

(50) $$\begin{array}{c} \underset{k\to \infty }{\lim}||Au_{k}-Bv_{k}||=0. \end{array}$$

Therefore, we have

(51) $$\begin{array}{c} \underset{k\to \infty }{\lim}||Ax_{k}-By_{k}||=0. \end{array}$$

Next we show that the sequence (*x*_*k*_, *y*_*k*_) generated by Algorithm 10 weakly converges to a solution of the SEP (1). Let $\left(\hat{x},\hat{y})\in \omega_{w}(x_{k},y_{k}\right)$; then there exist the two subsequences (*x*_*k*_*l*__) and (*y*_*k*_*l*__) of (*x*_*k*_) and (*y*_*k*_) which converge weakly to $\hat{x}$ and $\hat{y}$, respectively. The weak convergence of (*Ax*_*k*_*l*__ − *By*_*k*_*l*__) to $A\hat{x}-B\hat{y}$ and the lower semicontinuity of the squared norm imply that

(52) $$\begin{array}{c} ||A\hat{x}-B\hat{y}||\leq \underset{l\to \infty }{\mathrm{liminf}}||Ax_{k_{l}}-By_{k_{l}}||=0; \end{array}$$

that is, $A\hat{x}=B\hat{y}$.

Since *u*_*k*_*l*__ ∈ *C*_*k*_*l*__, we have

(53) $$\begin{array}{c} c\left(x_{k_{l}}\right)+\langle \xi_{k},u_{k_{l}}-x_{k_{l}}\rangle \leq 0. \end{array}$$

Thus

(54) $$\begin{array}{c} c\left(x_{k_{l}}\right)\leq -\langle \xi_{k_{l}},u_{k_{l}}-x_{k_{l}}\rangle \leq \xi ||u_{k_{l}}-x_{k_{l}}||, \end{array}$$

where *ξ* satisfies ||*ξ*_*k*_|| ≤ *ξ* for all *k* ∈ *ℕ*. The lower semicontinuity of *c* and the first formula of (49) lead to

(55) $$\begin{array}{c} c\left(\hat{x}\right)\leq \underset{l\to \infty }{\mathrm{liminf}}c\left(x_{k_{l}}\right)\leq 0, \end{array}$$

and therefore $\hat{x}\in C$.

Likewise, since *v*_*k*_*l*__ ∈ *Q*_*k*_*l*__, we have

(56) $$\begin{array}{c} q\left(y_{k_{l}}\right)+\langle \eta_{k_{l}},v_{k_{l}}-y_{k_{l}}\rangle \leq 0. \end{array}$$

Thus

(57) $$\begin{array}{c} q\left(y_{k_{l}}\right)\leq -\langle \eta_{k_{l}},v_{k_{l}}-y_{k_{l}}\rangle \leq \eta ||v_{k_{l}}-y_{k_{l}}||, \end{array}$$

where *η* satisfies ||*η*_*k*_|| ≤ *η* for all *k* ∈ *ℕ*. Again, the lower semicontinuity of *q* and the second formula of (49) lead to

(58) $$\begin{array}{c} q\left(\hat{y}\right)\leq \underset{l\to \infty }{\mathrm{liminf}}q\left(y_{k_{l}}\right)\leq 0, \end{array}$$

and therefore $\hat{y}\in Q$. Hence $(\hat{x},\hat{y})\in \Gamma$.

Following the same argument of Theorem 9, we can show the uniqueness of the weak cluster points and hence the whole sequence (*x*_*k*_, *y*_*k*_) weakly converges to a solution of the SEP (1), which completes the proof.

## References

[1] Moudafi A Alternating CQ-algorithm for convex feasibility and split fixed-point problems.

[2] Attouch H, Cabot A, Frankel P, Peypouquet J (2011). Alternating proximal algorithms for linearly constrained variational inequalities: application to domain decomposition for PDE’s. *Nonlinear Analysis: Theory, Methods & Applications*.

[3] Attouch H, Bolte J, Redont P, Soubeyran A (2008). Alternating proximal algorithms for weakly coupled convex minimization problems. Applications to dynamical games and PDE’s. *Journal of Convex Analysis*.

[4] Censor Y, Bortfeld T, Martin B, Trofimov A (2006). A unified approach for inversion problems in intensity-modulated radiation therapy. *Physics in Medicine and Biology*.

[5] Attouch H (2009). *Alternating Minimization and Projection Algorithms. From Convexity to Nonconvexity*.

[6] Chen R, Li J, Ren Y (2013). Regularization method for the approximate split equality problem in infinite-dimensional Hilbert spaces. *Abstract and Applied Analysis*.

[7] Byrne C, Moudafi A Extensions of the CQ algorithm for the split feasibility and split equality problems.

[8] Censor Y, Elfving T (1994). A multiprojection algorithm using Bregman projections in a product space. *Numerical Algorithms*.

[9] Dong QL, He S Solving the split equality problem without prior knowledge of operator norms.

[10] Tseng P (2000). Modified forward-backward splitting method for maximal monotone mappings. *SIAM Journal on Control and Optimization*.

[11] Zhao J, Yang Q (2012). Several acceleration schemes for solving the multiple-sets split feasibility problem. *Linear Algebra and Its Applications*.

[12] Pascali D, Sburlan S (1978). *Nonlinear Mappings of Monotone Type*.

[13] Yang Q (2004). The relaxed CQ algorithm solving the split feasibility problem. *Inverse Problems*.

[14] Dong QL, Yao Y, He S (2013). Weak convergence theorems of the modified relaxed projection algorithms for the split feasibility problem in Hilbert spaces. *Optimization Letters*.

[15] Fukushima M (1986). A relaxed projection method for variational inequalities. *Mathematical Programming*.

[16] Moudafi A (2013). A relaxed alternating CQ-algorithm for convex feasibility problems. *Nonlinear Analysis: Theory, Methods & Applications*.

